# Molecular epidemiology and drug resistant mechanism in carbapenem-resistant *Klebsiella pneumoniae* isolated from pediatric patients in Shanghai, China

**DOI:** 10.1371/journal.pone.0194000

**Published:** 2018-03-20

**Authors:** Xingyu Zhang, Di Chen, Guifeng Xu, Weichun Huang, Xing Wang

**Affiliations:** 1 Department of Laboratory Medicine, Shanghai Children's Medical Center, Shanghai Jiaotong University School of Medicine, Shanghai, P.R. China; 2 Department of Ophthalmology, The Second People's Hospital of Foshan, Guangdong, P.R. China; 3 Center for Drug Safety Evaluation and Research, Shanghai University of Traditional Chinese Medicine, Shanghai, P.R. China; Northwestern University Feinberg School of Medicine, UNITED STATES

## Abstract

Infection by carbapenem-resistant *Klebsiella pneumoniae* (CR-KP) is a public health challenge worldwide, in particular among children, which was associated with high morbidity and mortality rates. There was limited data in pediatric populations, thus this study aimed to investigate molecular epidemiology and drug resistant mechanism of CR-KP strains from pediatric patients in Shanghai, China. A total of 41 clinical CR-KP isolates from sputum, urine, blood or drainage fluid were collected between July 2014 and May 2015 in Shanghai Children's Medical Center. Multilocus sequence typing (MLST), antibiotic susceptibility testing, PCR amplification and sequencing of the drug resistance associated genes were applied to all these isolates. MLST analysis revealed 16 distinct STs identified within the 41 isolates, among which the most frequently represented were ST11(19.5%),ST25(14.6%),ST76(14.6%),ST37(9.8%).One new ST was first identified. All CR-KP isolates showed MDR phenotypes and were resistance to ceftazidime, imipenem, piperacillin / tazobactam, ceftriaxone, ampicillin /sulbactam, aztreonam. They were confirmed as carbapenemase producer, NDM-1 (56.1%, 23/41), IMP (26.8%, 11/41), KPC-2 (22.0%, 9/41) were detected. Of note, two isolates carried simultaneously both NDM-1 and IMP-4. All CR-KP strains contained at least one of extended spectrum β-lactamase genes tested(TEM, SHV, OXA-1, CTX-M group) and six isolates carried both ESBL and AmpC genes(DHA-1). Among the penicllinase and β-lactamase genes, the most frequently one is SHV(92.7%,38/41), followed by TEM-1(68.3%,28/41), CTX-M-14(43.9%,18/41), CTX-M-15(43.9%,14/41), OXA-1(14.6%,6/41). In the present study, NDM-1-producing isolates was the predominant CR-KP strains in children, follow by IMP and KPC-producing strains. NDM-1and IMP-4 were more frequent than KPC-2 and showed a multiclonal background. Those suggested carbapenem-resistant in children is diverse, and certain resistance mechanisms differ from prevalent genotypes in adults in the same region. Knowledge of the molecular epidemiology and drug resistant mechanism of CR-KP can have a profound effect on clinical treatment, infection control measures and public health policies for children.

## Introduction

*Klebsiella pneumoniae* is one of the most common Enterobacteriaceae associated with community- and hospital-acquired infections. In recent years, the misuse and overuse of antibiotics has given rise to the serious outcome worldwide[1], and infections caused by carbapenem-resistant *K*. *pneumoniae*(CR-KP) has been increasing rapidly with high rates of treatment failure and mortality[2]. According to CHINET, one of the largest antimicrobial resistance surveillance networks in China, carbapenem resistance rates among *K*. *pneumoniae* were 0.7% in 2000 and 2.9% in 2009[3], whereas the resistance rates rapidly increased to 14% in 2015. CR-KP was usually characterized by by multiantibiotic resistance which involves penicillins, all cephalosporins, monobactams, carbapenems, and even β-lactamase inhibitors. Therefore, the rapid dissemination of CR-KP isolates has become a serious public health problem.

Resistance of *K*. *pneumoniae* to carbapenems is related to several different mechanisms, in particular the production of strong carbapenemases, but also of beta-lactamases that harbors weak carbapenemase activity in combination with membrane impermeability[4]. It is known that several carbapenemases including KPC, NDM-1[5], VIM, IMP and OXA-48 were responsible for nonsusceptibility to carbapenems, without additional permeability defects[6]. These enzymes are encoded by genes in either chromosome or acquired mobile elements such as plasmids and transposons. KPC enzymes are currently the widest disseminated enzymes among *K*. *pneumoniae* isolates in Asia, America and Europe. More than 20 different KPC variants have been identified, among which KPC-2 and -3 were the most common types[7]. In China, carbapenem resistance has mainly resulted from the widespread of *Klebsiella pneumoniae* carbapenemase (KPC) and KPC-2 is the most common carbapenemase[8]. In addition, IMP, VIM and NDM have been reported to take part in carbapenem resistance of *K*. *pneumoniae* in China[9].

CR-KP isolates were usually multidrug resistant accompanied by resistance to extended-spectrum cephalosporins, fluoroquinolones and aminoglycosides. There were previous studies frequently described the co-prevalence of carbapenem resistance and extended spectrum beta-lactamase (ESBL) production[10]. Recently, the co-existence of 16S rRNA methylase, which can render enterobacteriaceae resistant to aminoglycoside, has also been reported in KPC-producing pathogens[11,12]. The potential spread of multiresistant *K*. *pneumoniae* would strongly limit the therapeutic options.

To better combat CR-KP infections, it is essential to understand the composition and distribution of antibiotic resistance genotypes. In addition, previous studies have mainly focused on adult populations, leaving a significant lacuna in pediatric populations. In the current study, we aim to investigate antimicrobial resistance profiles and co-existence of resistance determinants in CR-KP isolates from children and to assess the epidemiological relatedness of these isolates in Shanghai, China.

## Materials and methods

### Bacterial isolates

From July 2014 to May 2015, a total of 41 non-duplicated carbapenem-resistant *K*. *pneumoniae* strains collected from Shanghai Children Medical Center were selected for analysis. Shanghai Children's Medical Center is one of the largest pediatric hospitals in China with 800 beds and approximately 3,000 hospital admissions per day. All isolates were initially identified by colony morphology, biochemical testing, and VITEK 2 GN ID cards (bioMe´rieux, Inc.). 41 clinical isolates were recovered from sputum(n = 32), urine(n = 7), blood(n = 1) and drainage fluid(n = 1), respectively.

The Ethics Committee of Shanghai Children's Medical Center exempted this study from review because the present study focused on bacteria.

### Antimicrobial susceptibility testing

All isolates were processed with standard Laboratory procedures. Carbapenem resistance of *K*. *pneumoniae* was screened using the meropenem and imipenem disk. The antibiotic susceptibility of all isolates in this study was performed using the bioMe´rieux VITEK2 system and the AST-GN card following manufacturer’s instructions. Results were interpreted in accordance with Clinical and Laboratory Standards Institute (CLSI) guidelines (CLSI, 2014). The following 17 drugs were tested: ertapenem, amikacin, ceftazidime, imipenem, piperacillin / tazobactam, ceftriaxone, ciprofloxacin, trimethoprim-sulfamethoxazole (SXT), nitrofurantoin, ampicillin, tobramycin, cefazolin, levofloxacin, gentamicin, ampicillin /sulbactam, aztreonam, cefotetan. *Escherichia coli* ATCC 25922 and *Pseudomonas aeruginosa* ATCC 27853 were used as reference strains for susceptibility testing.

### MLST analysis

Isolates were screened according to the protocol described on the *K*. *pneumoniae* MLST website (http://www.pasteur.fr/recherche/genopole/PF8/mlst/Kpneumoniae.html)) to detect the following seven housekeeping genes: *gapA*, *infB*, *mdh*, *pgi*, *phoE*, *rpoB* and *tonB*. The sequences of the PCR products were compared with the existing alleles available from the MLST website, and the allelic number[sequence type (ST)] was determined for each sequence. Clustering of related STs, which were defined as clonal complexes (CCs), was determined using eBURST (based on related STs).

### Detection of extended-spectrum β-lactamase (ESBL) and plasmid-mediated AmpC β-lactamase genes

All original isolates were screened by multiplex PCRs with specific primers and conditions as previously described[13]. Specifically, a *bla*_TEM_/*bla*_SHV_/*bla*_OXA-1_-like multiplex PCR; a *bla*_CTX-M_ multiplex PCR including phylogenetic groups 1, 2 and 9; a plasmid-mediated AmpC β-lactamase gene multiplex PCR including six groups based on percentage of similarity; a *bla*_VEB_/*bla*_GES_/*bla*_PER_ multiplex PCR; and two carbapenemase gene multiplex PCRs, one including *bla*_VIM_, *bla*_IMP_ and *bla*_KPC_ genes and the other *bla*_GES_ and *bla*_OXA-48_-like genes. One simplex PCR was also designed for *bla*_CTX-M-8/-25._The PCR assay of *bla*_*NDM*_ was performed according to the previous protocol[14]

All of the positive PCR products were sequenced with an ABI3730 sequencer (Applied Biosystems) and the sequences were compared with the reported sequences from GenBank by Blast (www.ncbi.nlm.nih.gov/blast/).

### Detection of aminoglycoside-resistant and quinolone resistant genes

All original isolates were screened by PCR with specific primers for 16S rRNA methylase genes(*armA*, *rmtA*, *rmtB*, *rmtC* and *rmtD*)[15,16] belong to aminoglycoside-resistance and plasmid-mediated quinolone-resistant genes [*qnrA*, *qnrB*, *qnrC*, *qnrD*, *qnrS*, *aac(6’)-Ib-cr*, *qepA* and *oqxAB*][17]. All of the positive PCR products were sequenced analyzed as mentioned above.

## Results

### Clinical features

41 CR-KP isolates were collected from patients 4 days to 7 years old with local or systemic infection. Among them, twenty-two patients (65.9%) were male and fourteen patients (34.1%) were female. 92.7% (38/41) of the children were less than 1-year old and 9 were neonates (22.0%). From their medical records, respiratory infection was the most common infection type caused by CR-KP; 78.0% (32/41) of the isolates were from the respiratory tract, and 17.1% (7/41) of the isolates were associated with urinary tract infections. Co-morbidities included hematologic,genetic and respiratory abnormalities. Among the 41 patients, 26 had congenital heart disease and 9 had other complications such as aplastic anemia, pulmonary hypertension and respiratory failure. The majority of these patients typically had invasive devices. There had been no identified CR-CP outbreaks at our center during the study period. There was no direct evidence of epidemiologic relatedness between any two case.

### Antimicrobial susceptibility testing

The antimicrobial resistance profiles of 41 CR-KP isolates are listed in Table 1. All CR-KP isolates showed multi-resistance to antibiotics tested. For example, all strains were resistance to ertapenem, ceftazidime, imipenem, piperacillin / tazobactam, ceftriaxone, ampicillin, cefazolin, ampicillin /sulbactam, aztreonam and cefotetan. However, the resistance rates to other antimicrobials tested were not high, such as 24.4% to amikacin, 24.4% to levofloxacin, 26.8% to ciprofloxacin, 29.3% to tobramycin, 36.6% to trimethoprim-sulfamethoxazole (SXT), 43.9% to gentamicin and 58.5% to nitrofurantoin. The resistance profiles of CR-KP isolates differed by STs(Table 2). The ST11 strains had significantly higher multiple antibiotic-resistance profiles when compared with other STs. Most of the ST11 isolates exhibited resistance to ciprofloxacin, tobramycin, levofloxacin and gentamicin, while ST76 and ST25 isolates showed susceptible to ciprofloxacin, tobramycin, levofloxacin and gentamicin.

**Table 1:** Antimicrobial susceptibility of the 41 CR-KP isolates to 17 common antimicrobial agents.

Antibiotic	Antibiotic susceptibility (%)			MIC (μg/ml) (CLSI,2014)		
	S	I	R	S	I	R
Ertapenem	0	0	100	≤0.5	1	≥2
Amikacin	75.6	0	24.4	≤16	32	≥64
Ceftazidime	0	0	100	≤4	8	≥16
Imipenem	0	0	100	≤1	2	≥4
Piperacillin / tazobactam	0	0	100	≤16/4	32/4~64/4	≥128/4
Ceftriaxone	0	0	100	≤1	2	≥4
Ciprofloxacin	73.2	0	26.8	≤1	2	≥4
Trimethoprim-sulfamethoxazole (SXT)	63.4	0	36.6	≤2/38	—	≥4/76
Nitrofurantoin	7.3	34.2	58.5	≤32	64	≥128
Ampicillin	0	0	100	≤8	16	≥32
Tobramycin	53.7	17	29.3	≤4	8	≥16
Cefazolin	0	0	100	≤2	4	≥8
Levofloxacin	73.2	2.4	24.4	≤2	4	≥8
Gentamicin	53.7	2.4	43.9	≤4	8	≥16
Ampicillin /sulbactam	0	0	100	≤8/4	16/8	≥32/16
Aztreonam	0	0	100	≤4	8	≥16
Cefotetan	0	0	100	≤16	32	≥64

**Table 2:** Antimicrobial resistance of 41 CR-KP isolates against antimicrobial agents among different sequence type.

Sequence type	Isolates	Amikacin (IR%)	Ciprofloxacin (IR%)	Trimethoprim-sulfamethoxazole (SXT),(IR%)	Nitrofurantoin (IR%)	Tobramycin (IR%)	Levofloxacin (IR%)	Gentamicin (IR%)
ST11	8	100	87.5	37.5	100	100	100	100
ST25	6	0	16.7	16.7	100	16.7	16.7	16.7
ST76	6	0	16.7	66.7	100	0	16.7	16.7
ST37	4	25	0	75	100	75	0	50
ST17	3	0	0	33.3	66.7	0	0	33.3
ST45	2	0	0	0	33.3	0	0	0
ST334	2	0	0	0	0	100	0	100
ST20	2	0	0	0	100	0	0	0
ST278	1	0	0	0	100	100	0	100
ST414	1	0	0	0	100	0	0	0
ST147	1	0	100	100	100	0	0	0
ST1198	1	0	0	100	100	0	0	0
ST14	1	0	0	0	100	100	0	100
ST1699	1	0	100	100	100	100	100	0
ST1822	1	100	0	0	100	100	0	100
SLV of ST156	1	0	0	0	100	100	0	100

I,intermediate;R,resistant;IR, intermediate and resistant.

### MLST analysis

**Fig 1:**
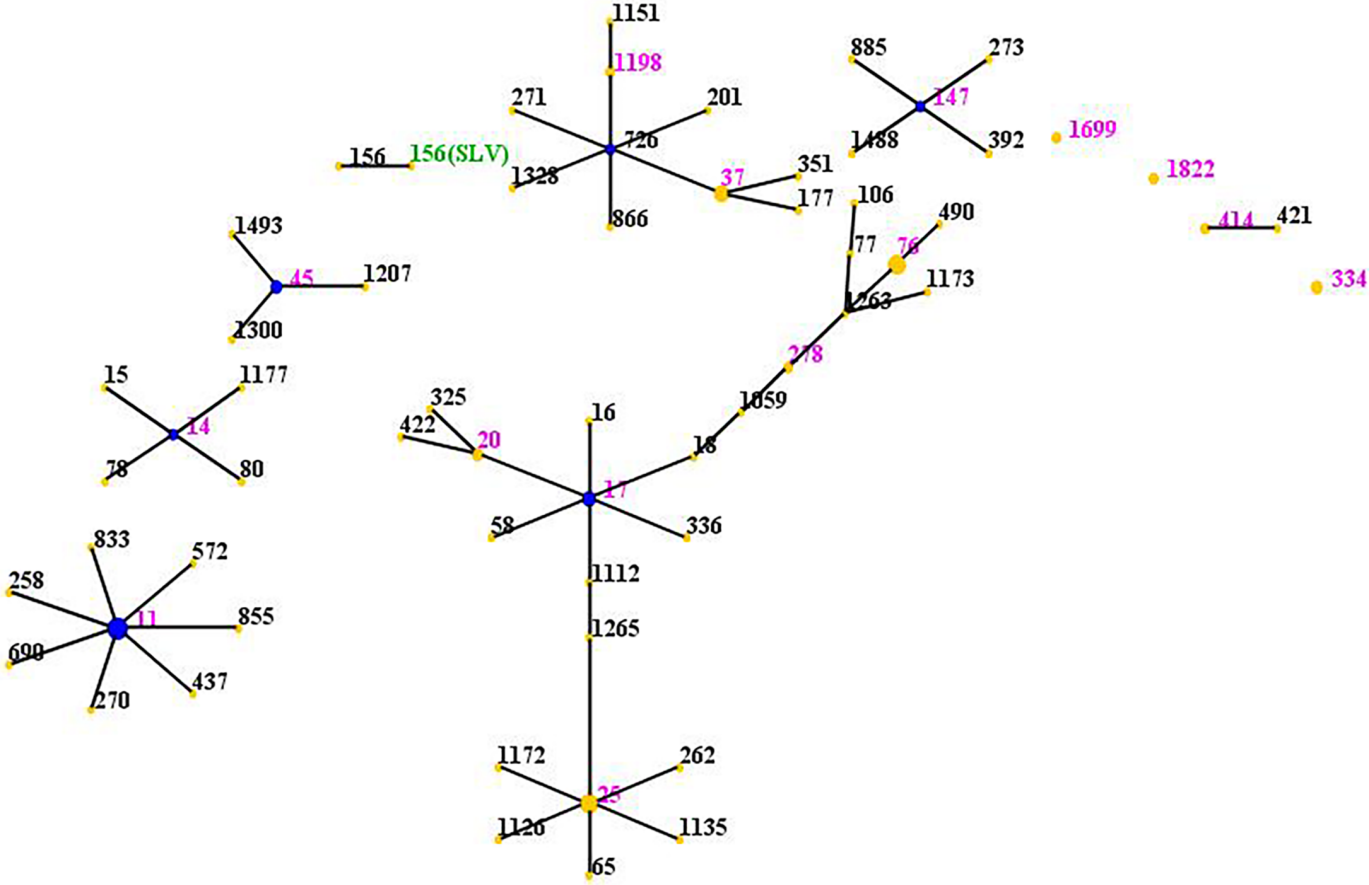
Distribution of STs in the clonal complexes. The eBURST application of the MLST data from all of the isolates analyzed in this study. The purple and green numbers represent 16 STs which are found in 41 CR-KP isolates. STs that are linked by a line belong to the same cluster. Circle sizes are proportional to the number of strains within the ST.

The genetic diversity of *a*ll CR-KP isolates was analyzed by MLST (Fig 1). The diversity index was 39.0% (16/41). Specially, there were 16 distinct STs identified within the 41 isolates. The most frequently represented STs were ST11 (19.5%), ST25 (14.6%), ST76 (14.6%), which was in accordance with previous studies that ST11 was the dominant clone of KPC-producing *Klebsiella pneumoniae* in China [8]. In addition, we found one new type, which is a single-locus variant of ST156. The other types are ST37(9.8%), ST17(7.3%), ST45(4.9%), ST334(4.9%), ST20((4.9%), ST278(2.4%), ST414 (2.4%), ST147 (2.4%), ST1198(2.4%), ST14(2.4%), ST1699(2.4%), ST1822(2.4%).

The eBURST analysis of CR-KP isolates using all STs available in the MLST database is shown (Fig 1). The eBURST algorithm clustered all 16 STs isolated from clinical patients into 7 CCs (CC11, CC25, CC17, CC76, CC37, CC45, CC334) and eight singletons.

### Distribution of ESBL and plasmid-mediated AmpC β-lactamase genes

PCR and sequencing analysis were conducted for 41 CR-KP isolates to detect ESBL and AmpC β-lactamase genes. The results showed that all strains contained ESBL genes (TEM, SHV, OXA-1, CTX-M group) and six isolates carried both ESBL and AmpC genes(DHA-1). Isolates often carried three, occasionally two or four, different types of broad-spectrum β-lactamase (TEM-1, SHV-1, SHV-11, OXA-1, CTX-M-14 or CTX-M-15). Among the β-lactamase genes, the most frequently one is SHV(92.7%,38/41),followed by TEM-1(68.3%,28/41), CTX-M-14(43.9%,18/41), CTX-M-15(43.9%,14/41) and OXA-1(14.6%,6/41).For the targeted carbapenemase genes, KPC-2 (22.0%,9/41),IMP (26.8%,11/41), NDM-1 (56.1%,23/41) were detected among the strains. Two isolates belonged ST334 carried both NDM-1 and IMP-4. DHA-1 (14.6%, 6/41) enzymes were the only observed plasmid mediated AmpC β-lactamases in the present study. Among the six isolates producing both an ESBL and a plasmid-mediated AmpC β-lactamase, six strains were found to co-produce DHA-1 and SHV. A complete description of the β-lactamases characterized in the 41 CR-KP isolates is available in Table 3.

**Table 3:** Drug resistance gene distribution among the molecular types of 41 CR-KP isolates from pediatric patients.

Sequence type	Isolates	Carbapenemase	Penicllinases and Extended-spectrum β-lactamase	AmpC β-lactamase	16S rRNA methylase	PMQR genes
ST11	8	KPC-2(8)	TEM-1(8),SHV11(8),CTX M-14(8)		*rmtB*(8)	*oqxAB*(5)
ST25	6	NDM-1(6)	TEM-1(6),SHV11(6),CTX M-15(6)			*oqxAB*(6)
ST76	6	NDM-1(6)	TEM-1(2),SHV11(6),CTX M-14(2)	DHA-1(4)	*armA*(1)	*aac*(1),*oqxAB*(6),*qnrB4*(4)
ST37	4	IMP-4(2),NDM-1(2)	TEM-1(1),SHV11(4),CTX M-15(1),CTX M-14(4)			*aac*(2),*oqxAB*(4),*qnrS1*(3)
ST17	3	NDM-1(3)	TEM-1(2),SHV11(3),CTX M-15(1),CTX M-14(1)	DHA-1(1)		*oqxAB*(4),*qnrS1*(1),*qnrB4*(1)
ST45	2	KPC-2(1),NDM-1(1)	SHV1(1),SHV11(1),CTX M-15(1)			*oqxAB*(2)
ST334	2	IMP-4(2),NDM-1(2)	TEM-1(2),OXA-1(2)			*aac*(2)
ST20	2	IMP-4(2)	TEM-1(1),SHV11(2),CTX M-15(1),CTX M-14(1)			*aac*(2),*oqxAB*(2),*qnrS1*(2)
ST278	1	IMP-4(1)	TEM-1(1),SHV27(2),OXA-1(1)			*aac*(1),*oqxAB*(1)
ST414	1	NDM-1(2)	TEM-1(1),SHV11(1),CTX M-15(1)			
ST147	1	IMP-4(1)	TEM-1(1),SHV11(1),CTX M-15(1)			*oqxAB*(1)
ST1198	1	NDM-1(1)	SHV11(1),CTX M-14(1)	DHA-1(1)		*oqxAB*(1),*qnrS*(1),*qnrB4*(1)
ST14	1	IMP-4(1)	TEM-1(1),SHV1(1),OXA-1(1),CTX M-15(1)			*aac*(1),*oqxAB*(1)
ST1699	1	NDM-1(1)	SHV11(1),OXA-1(1)			*aac*(1),*oqxAB*(1),*qnrS1*(1)
ST1822	1	IMP-8(1)	TEM-1(1),CTX M-15(1)		*armA*(1)	*aac*(1),*oqxAB*(1)
SLV of ST156	1	IMP-4(1)	TEM-1(1),SHV11(1),OXA-1(1)			*aac*(1),*oqxAB*(1)

aac: aac(6’)-Ib-cr

PMQR: plasmid-encoded quinolone resistance-associated genes

### Distribution of aminoglycoside-resistant and quinolone resistant genes

Posttanscriptional methylation of 16S rRNA has been reported which results in high-level resistance to aminoglycoside antibiotics [18]. All original isolates were screened by PCR for five 16S rRNA methylase genes(*armA*, *rmtA*, *rmtB*, *rmtC* and *rmtD*) (Table 3), *armA* and *rmtB* were detected to be 4.9% and 19.5%, respectively. None of the strains carried *rmtA*, *rmtC* and *rmtD*. For quinolone resistance, all isolates were also screened for *oqxAB* and other plasmid-encoded quinolone (PMQR) resistance-associated genes [*qnrA*, *qnrB*, *qnrC*, *qnrD*, *qnrS*, *aac(6’)-Ib-cr*, and *qepA*]. The highest rates were observed for *oqxAB*(85.4%,35/41), follow by *aac(6’)-Ib-cr*(24.4%,10/41), *qnrS1*(19.5%,8/41), *qnrB4*(14.6%,6/41). None of the isolates carried *qnrA*, *qnrC*, *qnrD* or *qepA*. In addition, 14 isolates in this study were positive for two PMQR genes, while 4 isolates were positive for three PMQR genes.

### Relations between sequence types and drug resistance determinants of CR-KP isolates

Several associations were observed between the STs and different drug resistance determinants. We found, most of the ST11 isolates contained TEM-1, SHV-11, CTX-M-14, KPC-2, *oqxAB* and *rmtB*, whereas ST25 isolates carried TEM-1, SHV-11, CTX-M-15, NDM-1 and *oqxAB*, ST76 isolates included SHV-11, DHA-1, NDM-1, *oqxAB* and *qnrB4*.

There was a strong association observed between specific ST and carbapenemase genes. For example, KPC-2 was mostly found in the ST11 strains, whereas NDM-1 was mainly observed in ST25, ST76 and ST334 isolates. IMP-4 was detected in more different STs, including ST156, ST278, ST334, ST147, ST20 and ST14.ST334 was the sole one which produced both NDM-1 and IMP-4.

For 16S rRNA methylase genes, *rmtB* was exclusively observed in 8 ST11 strains and *armA* was found in ST37 and ST1465 isolates, only one each sequence type. Except ST334 and ST414, *oqxAB* exist in all other STs. *qnrB4* was mainly detected in ST76 isolates, whereas *qnrS1*was found in ST20, ST37, ST740 and ST1198.

## Discussion

Infections by Carbapenem-Resistant Enterobacteriaceae (CRE), in particular carbapenem-resistant *Klebsiella pneumoniae* (CR-KP), are a significant public health challenge. Most of published studies have focused largely on adult populations, creating a significant lacuna in pediatric populations. The aim of the present study was to investigate molecular epidemiology and drug resistant mechanism of CR-KP isolates from pediatric patients between July 2014 and May 2015 in Shanghai, China.

In China, ST11 was reported to be the predominant clone of KPC-producing *K*. *pneumoniae* and KPC-2 is the most common carbapenemase in *K*. *pneumoniae*[8]. In accordance with previous data, ST11(19.5%) was also found to be the most prevalent clone in our study. ST11 is a single-locus variant (*tonB*) of ST258, the latter one was reported as a well known dominant molecular epidemiology clone worldwide, especially in the USA [19]. They belonged to CC258, which spread rapidly across the world. Except ST11, ST25 (14.6%), ST76 (14.6%), ST37 (9.8%) and other STs were found in the present study and the diversity index was 39.0% (16/41), indicating CR-KP had more diverse genetic backgrounds among children than adult [20]. It is essential to pay more attention on this problem from the public health point of view.

Except class A carbapenemases KPC, class B metallo-beta-lactamases (MBLs),including IMP, VIM, NDM and class D carbapenem-hydrolysing oxacillinase-48 (OXA-48)also have been reported to the most commonly identified carbapenemases worldwide. For those targeted carbapenemase genes, KPC-2(22.0%, 9/41), IMP (26.8%, 11/41), NDM-1 (56.1%,23/41) were detected in the present study. Recently, there were two reports indicating the rapid spread of bla_NDM-1_-producing *K*. *pneumoniae* in neonates, one was about two outbreaks caused by bla_NDM-1_-producing *K*. *pneumoniae* ST17 and ST20 in Shandong[21], the other one was a nosocomial outbreak of bla_NDM-1_-producing *K*. *pneumoniae* ST37 and ST76 in Shanghai[22]. ST37, ST76, ST17 and ST20 were found in the present study and major producer (36.6%) of NDM-1 carbapenemases. Moreover, the dissemination of bla_IMP-4_-producing *K*. *pneumoniae* has been increasing[23,24]. NDM-1and IMP-4 were more frequent than KPC-2 and showed a multiclonal background. All KPC-2 producing strains were of ST11, except for one isolate with ST45, whereas NDM-1 and IMP-4 producing strains had different clonal backgrounds. NDM-1 were mainly observed in ST25, ST76,ST37 and ST334 isolates, and IMP4 were detected in more different STs, including ST156,ST278,ST334, ST147, ST20 and ST14. From this, we speculated that NDM-1 and IMP-4 existed in genetic elements of good mobilization, such as plasmids, transposons and insertion sequences, which easily to be captured and transmitted by different clones.

In the present study, the phenomenon that the coexistence of multiple drug resistance determinants in single CR-KP strain was very common[10,25]. This is the reason why clinical strains were resistant to many kinds of drug at the same time. But we also found different ST seemed to carry different drug resistant profiles. Most of the ST11 isolates exhibited resistance to ciprofloxacin, tobramycin, levofloxacin and gentamicin, while ST76 and ST25 isolates showed susceptible to ciprofloxacin, tobramycin, levofloxacin and gentamicin. These phenotypes were proved by the corresponding drug resistance-associated genes they possessed. As a result, knowledge of the molecular epidemiology and drug resistant mechanism of CR-KP can provide a clue to clinical treatment and infection control measures for children.

## Funding

This study was supported by the National Natural Science Foundation of China (grants 81301392) and the Training Program for Outstanding Young Teachers in Higher Education Institutions (ZZjdyx13132), the Training Program for Clinical Medical Young Talents in Shanghai (HYWJ201605), Visiting Scholar Research Program and SCMC-EPT Program to Xing Wang. The work was also supported by Foshan Key Medical Training Project(Fspy1- 2015005) and Foshan 13th Five-year Key and Charactcteristic Medical building Project(FSGSPZD-135020).
